# Lithium and cobalt co-doped mesoporous bioactive glass nanoparticles promote osteogenesis and angiogenesis in bone regeneration

**DOI:** 10.3389/fbioe.2023.1288393

**Published:** 2024-01-04

**Authors:** Xin Zhang, Kai Nan, Yuankai Zhang, Keke Song, Zilong Geng, Donglong Shang, Lihong Fan

**Affiliations:** ^1^ Department of Orthopaedics, The Second Affiliated Hospital of Xi’an Jiaotong University, Xi’an, Shaanxi, China; ^2^ Department of Orthopaedics, The Second Affiliated Hospital of Air Force Medical University, Xi’an, Shaanxi, China; ^3^ Department of Osteonecrosis and Joint Reconstruction Surgery, Honghui Hospital, Xi’an Jiaotong University, Xi’an, Shaanxi, China; ^4^ Department of Anesthesiology, The First Affiliated Hospital of Xi’an Jiaotong University, Xi’an, Shaanxi, China

**Keywords:** bone regeneration, mesoporous bioactive glass nanoparticles, lithium, cobalt, osteogenesis, angiogenesis

## Abstract

Healing of severe fractures and bone defects involves many complex biological processes, including angiogenesis and osteogenesis, presenting significant clinical challenges. Biomaterials used for bone tissue engineering often possess multiple functions to meet these challenges, including proangiogenic, proosteogenic, and antibacterial properties. We fabricated lithium and cobalt co-doped mesoporous bioactive glass nanoparticles (Li-Co-MBGNs) using a modified sol-gel method. Physicochemical analysis revealed that the nanoparticles had high specific surface areas (>600 m^2^/g) and a mesoporous structure suitable for hydroxyapatite (HA) formation and sustained release of therapeutic ions. *In vitro* experiments with Li-Co-MBGNs showed that these promoted angiogenic properties in HUVECs and pro-osteogenesis abilities in BMSCs by releasing Co^2+^ and Li^+^ ions. We observed their antibacterial activity against *Staphylococcus aureus* and *Escherichia coli*, indicating their potential applications in bone tissue engineering. Overall, our findings indicate the feasibility of its application in bone tissue engineering.

## 1 Introduction

Bone defects caused by trauma, tumors, infections, and genetic malformations have always been a significant challenge for clinicians (O'Keefe and Mao, 2011). Conventional bone graft strategies (e.g., autografts and allografts) frequently yield favorable therapeutic effects, but they have limitations such as insufficient donor tissue, morbidity, surgical complications, pathophoresis, and immunological rejection (Ho-Shui-Ling et al., 2018; Perez et al., 2018). Bone tissue engineering (BTE) has attracted increasing attention as an alternative bone-grafting method because of its tunable physicochemical properties and low immunogenicity (El-Rashidy et al., 2017). The purpose of BTE is to generate new bone tissue with normal function using a combination of biomaterials, bioactive molecules, and cells (Zhang et al., 2022a). Despite the remarkable progress of BTE, current biomaterials are still limited by their insufficient biological capabilities. Bone regeneration is a complex and intricate process that cannot be achieved by current biomaterials alone. Therefore, novel biomaterials with multiple biological properties are urgently needed.

Bioactive glasses (BGs) are promising biomaterials for BTE. They are mainly based on silica networks consisting of calcium and phosphate oxides, which endow them with osteoconductivity, biodegradability, mineralization, and interfacial bonding capabilities, making them suitable for bone fracture repair and bone tissue regeneration applications (Wu and Chang, 2014; Kurtuldu et al., 2021a). Mesoporous BGs (MBGs) combine the properties of conventional BGs with ordered mesoporous structures, exhibiting an enlarged specific surface area with better biodegradability and mineralization capability, making them ideal drug/growth factor carriers and paving the way for biomedical applications (Pontremoli et al., 2018; Kurtuldu et al., 2021b). Mesoporous BG nanoparticles (MBGNs) are nanosized, which further increases their specific surface area and makes them suitable bioactive fillers for composite biomaterials, suggesting that they can potentially be used in BTE (Kurtuldu et al., 2021a; Zhang et al., 2022b). In addition to the structural characteristics of MBGNs, whose components can be tailored to enhance their biological functions, various therapeutic ions have been incorporated into MBGNs, resulting in desirable biological properties (Wu and Chang, 2014; Westhauser et al., 2021a).

Therapeutic ions lithium (Li) and cobalt (Co) have attracted significant attention owing to their unique biological properties. Li can enhance the proliferation and osteogenic differentiation of bone marrow stromal cells (BMSCs), upregulate osteogenesis-related gene expression, and inhibit osteoclast growth and macrophage osteoclastogenesis, thereby promoting bone formation (Khorami et al., 2011; Han et al., 2012; Wu et al., 2014; Pan et al., 2018). Consistently, a clinical trial demonstrated that Li can preserve or enhance bone mass (Zamani et al., 2009). In addition, Li upregulates the expression of proangiogenic genes in BMSCs and human umbilical vein endothelial cells (HUVECs), promotes BMSC-EC communication, and ultimately enhances angiogenesis (Haro Durand et al., 2017; Liu et al., 2019). Li has been successfully incorporated into BGs and demonstrated pro-osteogenic and pro-angiogenic abilities *in vitro* and *in vivo* (Khorami et al., 2011; Wu and Chang, 2014; Wu et al., 2014; Miguez-Pacheco et al., 2016; Liu et al., 2019). Co has also attracted significant interest as another therapeutic ion widely used in orthopedics as a component of Co-based biomaterials such as the Co-28Cr-6Mo casting alloy, which is used in artificial joint prostheses. Co may provoke cytotoxicity or oxidative stress in cells at high concentrations, but it could positively affect the expression of vascular endothelial growth factor (VEGF) within adequate doses via the stabilization of protein level hypoxia-inducible factor 1-α (HIF-1α), which could subsequently enhance angiogenesis (Yuan et al., 2003; El-Fiqi and Kim, 2021; Laia et al., 2021). Co has been shown to yield excellent antibacterial effects against gram-positive and -negative bacteria while maintaining good biocompatibility at appropriate concentrations (Duan et al., 2022). Over the years, Co has been incorporated into various biomaterials, including BGs, and demonstrated excellent pro-angiogenic and antibacterial abilities *in vitro* and *in vivo* (Wu et al., 2012; Boldbaatar et al., 2019; Deng et al., 2019; Zheng et al., 2019). However, to the best of our knowledge, no studies have reported the fabrication of Li and Co co-doped MBGNs (Li-Co-MBGNs) or their combination to achieve synergism, although these two ions have become research hotspots in bone tissue engineering.

In this study, we aimed to simultaneously synthesize Li- and/or Co-doped MBGNs with multiple biological functions, including antibacterial activity and the stimulation of osteogenesis and angiogenesis. Additionally, their mesoporous structure may make them suitable carriers of therapeutic drugs/growth factors, or bioactive fillers for bone repair and regeneration applications. To this end, we adopted a sol-gel method using cetyltrimethylammonium bromide (CTAB) as a template reagent and directly added metallic precursors to the synthesis system during the synthesis process. The synthesized MBGNs were comprehensively characterized in terms of morphology, microstructure, composition, *in vitro* bioactivity, and dissolution behavior. We further evaluated the effects of Li and/or Co incorporation on bacterial growth and various *in vitro* biological responses of the cells, including cytocompatibility, osteogenesis, and angiogenesis.

## 2 Materials and methods

### 2.1 Nanoparticle synthesis

Li-Co-MBGNs were fabricated using the sol-gel method as described in previous studies (Xin et al., 2017; Zhang et al., 2018; Neščáková et al., 2019; El-Fiqi and Kim, 2021; Rahmani et al., 2021). Solution A was prepared by dissolving 1.5 g of CTAB (Aladdin, Shanghai, China) in Tris-HCl buffer solution (pH = 8.0) while agitating continuously at 60°C. Then, 13.22 mL of tetraethyl orthosilicate (TEOS) was added to solution A under constant agitation. Following that, 3.80 g of calcium nitrate tetrahydrate (Ca(NO_3_)_2_·4H_2_O), 1.16 mL of triethyl phosphate, 0.36 g lithium chloride, and 0.22 g cobalt chloride (CoCl_2_) were sequentially added to the above solution. All precursors were added at predetermined molar ratios. The clear solution progressively became opaque after 16 h of vigorous stirring because of the formation of a white precipitate. A white precipitate was obtained after 8 min of centrifugation at 11,000 rpm, followed by three cycles of alternate rinsing with deionized water and ethanol. The resulting powder was desiccated under vacuum at ambient temperature for 24 h and sintered in air at 650°C for 3 h (2°C min^−1^) to remove CTAB and organic components and obtain Li-Co-MBGNs. MBGNs, Li-MBGNs, and Co-MBGNs were fabricated using the same method but without Li and/or Co. The nominal compositions of the four types are listed in Supplementary Table S1. We focused on the synergistic effects of Li and Co on angiogenesis, osteogenesis, and antibacterial activity, and fixed doping conditions for Li and Co were selected based on preliminary results (Wu et al., 2012; Moghanian et al., 2017).

The un-doped, Li-doped, Co-doped, and Li-Co-doped MBGNs are denoted as uMBGNs, Li-MBGN, Co-MBGN, and Li-Co-MBGN, respectively, and all bioactive glass nanoparticles are hereafter denoted as MBGNs.

### 2.2 MBGN characterization

The morphology was examined using field-emission scanning electron microscopy (GeminiSEM 500, ZEISS, Germany) before and after immersion in simulated body fluid (SBF). Five mg of MBGNs were dispersed in 5 mL of ethanol. MBGN powder underwent a gold-coating process using a sputtering technique (carbon coater MC1000, Hitachi, Japan). Transmission electron microscopy (TEM) (Talos L120C, Thermo Scientific, Czech Republic) was used to characterize the morphology and structure. Phase composition, both prior to and subsequent to immersion in SBF, was determined by X-ray diffraction (XRD) (XRD-6100, Shimadzu, Japan), using Cu Kα radiation in the 2θ range of 10°–80°. The step size and dwell time were set at 0.010° and 1°/min, respectively. Fourier transform infrared spectroscopy (FTIR) spectra were recorded on a Perkin Elmer 100 serial spectrophotometer equipped with universal attenuated total reflectance (ATR) in the range of 400–4000 cm^-1^ with a resolution of 4.0 cm^-1^. N_2_ adsorption-desorption measurements were performed using an ASAP2460 instrument (Micromeritics, United States). Prior to analysis, samples were subjected to vacuum outgassing at 150°C for 5 h. The Brunauer–Emmett–Teller (BET) method was used to determine the surface area, and the Barrett-Joyner-Halenda (BJH) method was employed to calculate the pore size. Solid Nuclear Magnetic Resonance (NMR; AVANCEIII/WB-400, Switzerland) was used for ^29^Si MAS NMR spectroscopy, and the degree of condensation (Dc) was calculated using the following formula:

$$Dc=\frac{\left(Q^{1}\;\%+2\times Q^{2}\%+3\times Q^{3}\%+4\times Q^{4}\%\right)}{4}$$

### 2.3 Bioactivity assays

MBGNs were immersed in SBF, and HA crystals formed on their surface at predetermined time points (Neščáková et al., 2019). SBF was prepared as described previously (KokuboT et al., 2006), and aliquots containing 75 mg of MBGNs were submerged in 50 mL of SBF. On days 3 and 7, scanning electron microscopy (SEM), Fourier transform infrared spectroscopy (FTIR), and X-ray diffraction (XRD) were used to assess surface HA layer formation.

### 2.4 Ion-release profiling

MBGNs were soaked in SBF (1.5 mg/mL). On days 1, 3, and 7, the supernatants were collected, and concentrated HNO_3_ was added to ensure complete dissolution. Ion concentrations were measured using ICP-OES (ICPS-9000, Shimadzu, Japan) after appropriate dilutions.

### 2.5 Antibacterial assays

The spread-plate method was used as described previously (Ding et al., 2021). The detailed steps are provided in the Supplementary Material.

### 2.6 Preparation of MBGN extracts

Elution tests were performed according to the International Standard Organization (ISO 10993-5). Samples at 1, 5, and 10 mg/mL concentrations were immersed in α-minimum essential media (α-MEM) and shaken at 37°C for 24 h, then clarified at 1800 rpm for 10 min, and the supernatant was collected and sterilized through a 0.22 μm filter. The culture medium supplemented with the extract was obtained using this process.

### 2.7 Cell culture

Human bone marrow stromal cells (hBMSCs) and human umbilical vein endothelial cells (HUVECs) were acquired from the China Center for Type Culture Collection (Wuhan University) and maintained at 37°C in a humidified 5% CO_2_ atmosphere in α-MEM (Sigma). To evaluate the effects of MBGNs on cellular behavior, α-MEM was replaced with conditioned medium containing MBGN extracts.

### 2.8 Cell proliferation assay

Proliferation was measured using a 3-(4,5-dimethylthiazol-2-yl)-2,5-diphenyltetrazolium bromide (MTT) Cell Proliferation and Cytotoxicity Assay Kit (Beyotime, Shanghai, China) following the manufacturer’s protocol. The detailed steps are provided in the Supplementary Material.

### 2.9 Cell adhesion assays

The MBGNs were pressed into bioactive glass tablets (BGTs) (5 mm diameter, 2 mm thickness) using a bolt press and hydraulic die. Prior to cell seeding, BGTs were sterilized at 120°C for 1.5 h. The hBMSCs were seeded onto the BGTs in α-MEM at a density of 1×10^4^ per well, followed by incubation under standard conditions (5% CO_2_ at 37°C) for 3 days. BGTs were washed with PBS, fixed with 2.5% glutaraldehyde in PBS for 2 h, and dehydrated in graded ethanol (50%, 70%, 90%, 95%, and 100%). Before the SEM observation, the dried BGTs were coated with a thin layer (approximately 10 nm) of gold via sputtering (Carboncoater MC1000, Hitachi, Japan) to make them conductive. The morphology of the attached cells on the surface was observed using SEM.

### 2.10 Osteogenic differentiation

Differentiation of hBMSCs was evaluated using alkaline phosphatase (ALP) staining, ALP activity detection, and alizarin red staining (ARS). ALP staining was performed as previously described (Wu et al., 2012) using an ALP staining kit (Beyotime, Shanghai, China). ALP activity was measured using an ALP Activity Assay Kit (Elabscience, Wuhan, China). ARS was performed using an osteoblast mineralized nodule staining kit (Beyotime, Shanghai, China). The detailed steps are provided in the Supplementary Material.

### 2.11 HUVEC angiogenesis

The effects of MBGN extracts on the angiogenic potential of HUVECs were assayed using tubule formation, wound healing, and transwell assays. The detailed steps are provided in the Supplementary Material.

### 2.12 Immunofluorescence

Cells were seeded on the glass slides in 12-well plates at a density of 1×10^5^ per well and incubated in 5% CO_2_ at 37°C overnight. The culture medium was replaced with a medium containing MBGN extracts, and the cells were cultured for another 3 days. The cells were then washed with PBS, fixed with 4% paraformaldehyde for 10 min, and permeabilized with 0.2% Triton X-100 for 10 min. Cells were blocked with 3% BSA for 30 min, incubated with primary antibodies against OCN, VEGF, and F-actin (Abcam, Cambridge, United States), followed by incubation with conjugated secondary antibodies (red fluorescent dye for OCN and VEGF, green for F-actin), and counterstaining with DAPI. Cells were imaged using fluorescence microscopy, and fluorescence intensities were measured using ImageJ software.

### 2.13 Quantitative real-time polymerase chain reaction (qRT-PCR)

The expression of mRNAs encoding osteogenic [ALP, OCN, osteopontin (OPN), and runt-related transcription factor 2 (RUNX-2)] on days 1, 3, and 7, as well as angiogenic (hypoxia-inducible factor 1-alpha (HIF-1α), VEGF, and kinase insert domain receptor (KDR)) markers on days 3 and 7, were measured as previously described (Zhu D. et al., 2021). The primers used are listed in Supplementary Tables S2, S3.

### 2.14 Statistical analysis

All results are expressed as means ± standard deviation (SD). Statistical analysis was performed with a one-way analysis of variance (ANOVA) with a Tukey test as a *post hoc* test. Analyses were conducted using the SPSS software (version 20.0; SPSS Inc., United States). All experiments were repeated at least three times; *p* < 0.05 was considered statistically significant.

## 3 Results

### 3.1 Characteristics of MBGNs

Photographs of MBGN powder are shown in Supplementary Figure S1. MBGNs and Li-MBGNs were white, whereas Co-MBGNs and Li-Co-MBGNs were light blue, likely because of d-d electronic transitions involving Co^2+^ ions ([Ar] 3d7), as described in a previous study (El-Fiqi and Kim, 2021).

**FIGURE 1:**
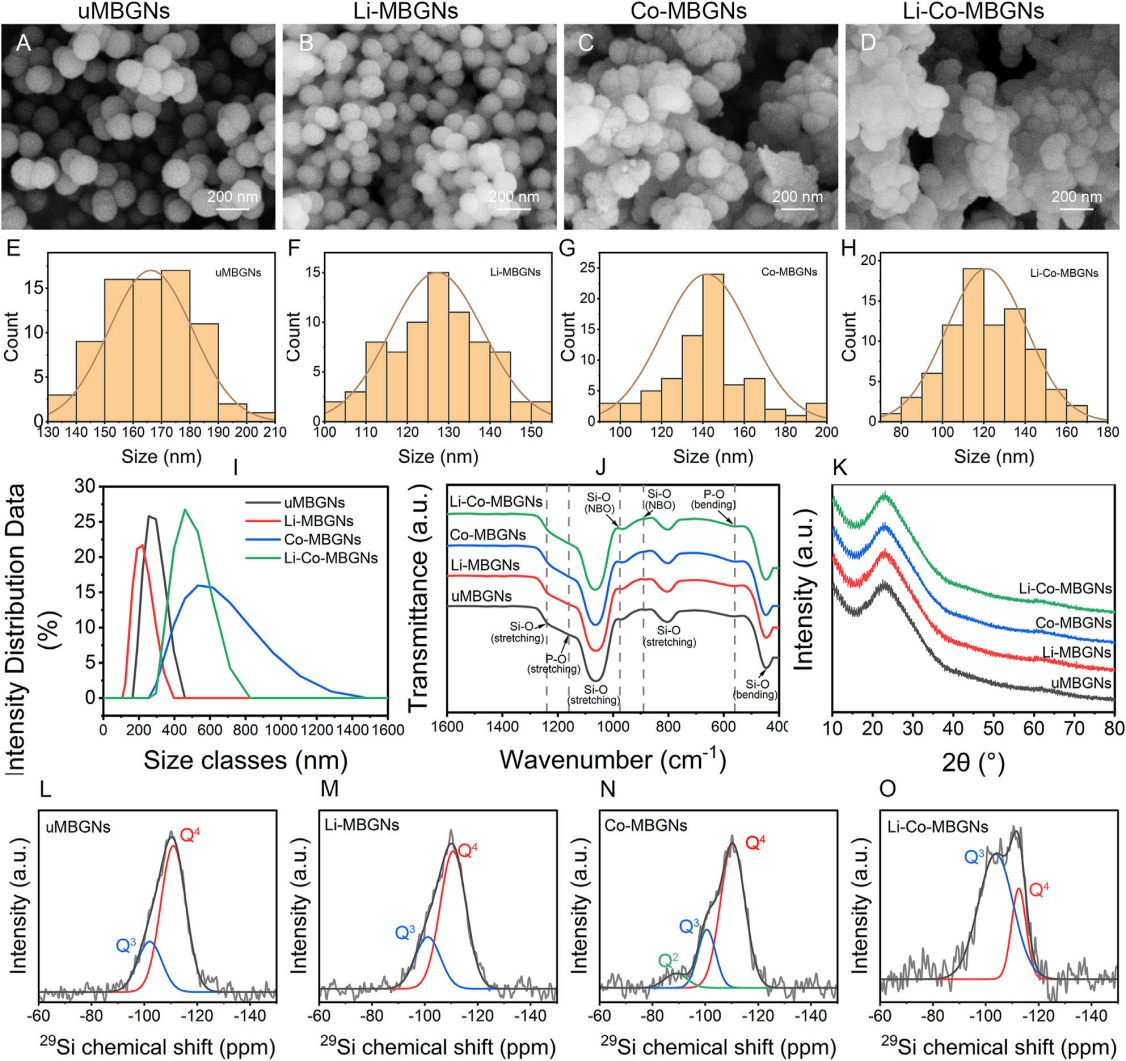
MBGN morphology and structure. **(A–D)** SEM images for uMBGNs **(A)**, Li-MBGNs **(B)**, Co-MBGNs **(C)**, and Li-Co-MBGNs **(D)**. **(E–H)** Size distributions measured from SEM images. Dynamic light scattering (DLS) spectra **(I)**, Fourier-transform infrared spectroscopy (FTIR) spectra **(J)**, and X-ray diffraction (XRD) patterns **(K)**. **(L–O)** ^29^Si MAS NMR spectra of uMBGNs **(L)**, Li-MBGNs **(M)**, Co-MBGNs **(N)**, and Li-Co-MBGNs **(O)**.

The microstructures of the samples were assessed by SEM. As shown in Figures 1A, B; Figures 1E, F, the MBGNs and Li-MBGNs had spherical shapes, good dispersion, and sizes of 166 ± 15 nm and 127 ± 11 nm, respectively. As shown in Figures 1C, D; Figures 1G, H, Co-MBGNs and Li-Co-MBGNs tended to aggregate and had irregular spherical shapes with sizes of 142 ± 21 nm and 121 ± 19 nm, respectively. Our findings reveal that the addition of Li to MBGNs resulted in a smaller particle size, which we attribute to a more compact silicate network, consistent with previous findings (Xin et al., 2020). In contrast, the addition of Co promoted MBGN aggregation.

DLS (Figure 1I; Supplementary Table S4) revealed that the Li-MBGNs were smaller than MBGNs, with sizes larger than those measured by SEM because of the silica-rich gel layer on the surface in contact with the solution (37). The sizes of the Co-MBGN and Li-Co-MBGN aggregates were 592 ± 9 nm and 454 ± 8 nm, respectively, indicating that ultrasound could disperse the aggregates.

The ζ-potentials of MBGNs, Li-MBGN, Co-MBGN, and Li-Co-MBGN were −31.5, −29.2, −24.2, and −23.8 mV, respectively (Supplementary Table S4). It has been reported that ζ-potential above ±30 mV prevent the aggregation of nanoparticles by repulsion, thereby increasing suspension stability (Luz and Mano, 2013; Vale et al., 2019). This is consistent with the good dispersion observed using SEM.

The chemical structures were examined by FTIR spectroscopy (Figure 1J)**.** All four MBGNs showed typical absorption bands for bioactive glass, including asymmetric Si–O–Si stretching vibrations at 1000–1250 cm^−1^, Si–O–Si bending vibrations at 800 cm^−1^, and Si–O–Si rocking vibrations at 450 cm^−1^, indicating the formation of silicate networks (Barrioni et al., 2018). The absorption bands at 890 and 975 cm^−1^ correspond to the non-bridging oxygen of the SiO_4_ tetrahedral structures, indicating that the metal cations act as network modifiers. We attribute the absorption bands at 1160 cm^−1^ and 500–600 cm^−1^ to the symmetric stretching and bending of P-O, respectively (Barrioni et al., 2018). The XRD spectra (Figure 1K) revealed the phase compositions of the four MBGNs. A broad peak over 15°–34° (2θ) with no major crystal peaks in any of the MBGNs indicated that they were amorphous and well-dispersed without the formation of metal and metal oxide nanoparticles.

The degree of polymerization and network connectivity of the silica network in BGs, which are related to both solubility and *in vitro* bioactivity, are primarily determined by Q^n^ species, where n is the number of bridging oxygen atoms and ranges from 0 to 4 (Zhang et al., 2022a). To investigate the species in MBGNs, ^29^Si MAS NMR was performed (Figures 1L–O); the resulting spectra were deconvoluted into three component curves around −110 ppm for Q^4^, −100 ppm for Q^3^, and -91 ppm for Q^2^ (Leonova et al., 2008; Eckert, 2018; Zhang et al., 2022a; Zhang et al., 2022b). The chemical shifts and relative fractions of the Q^n^ species are listed in Supplementary Table S5. All MBGNs contained Q^4^ and Q^3^ species as the primary components, whereas Q^2^ species were only detected in Co-MBGNs. Compared to uMBGNs, Li-MBGNs had a slightly lower abundance of Q^4^ species, whereas that of Co-MBGNs and Li-Co-MBGNs significantly decreased. Network connectivity was inversely proportional to the Q^4^ variation, indicating that Li and Co reduced both the polymerization and network connectivity of the silicate network. These results suggest that Li and Co in the MBGNs may act as network modifiers.

**FIGURE 2:**
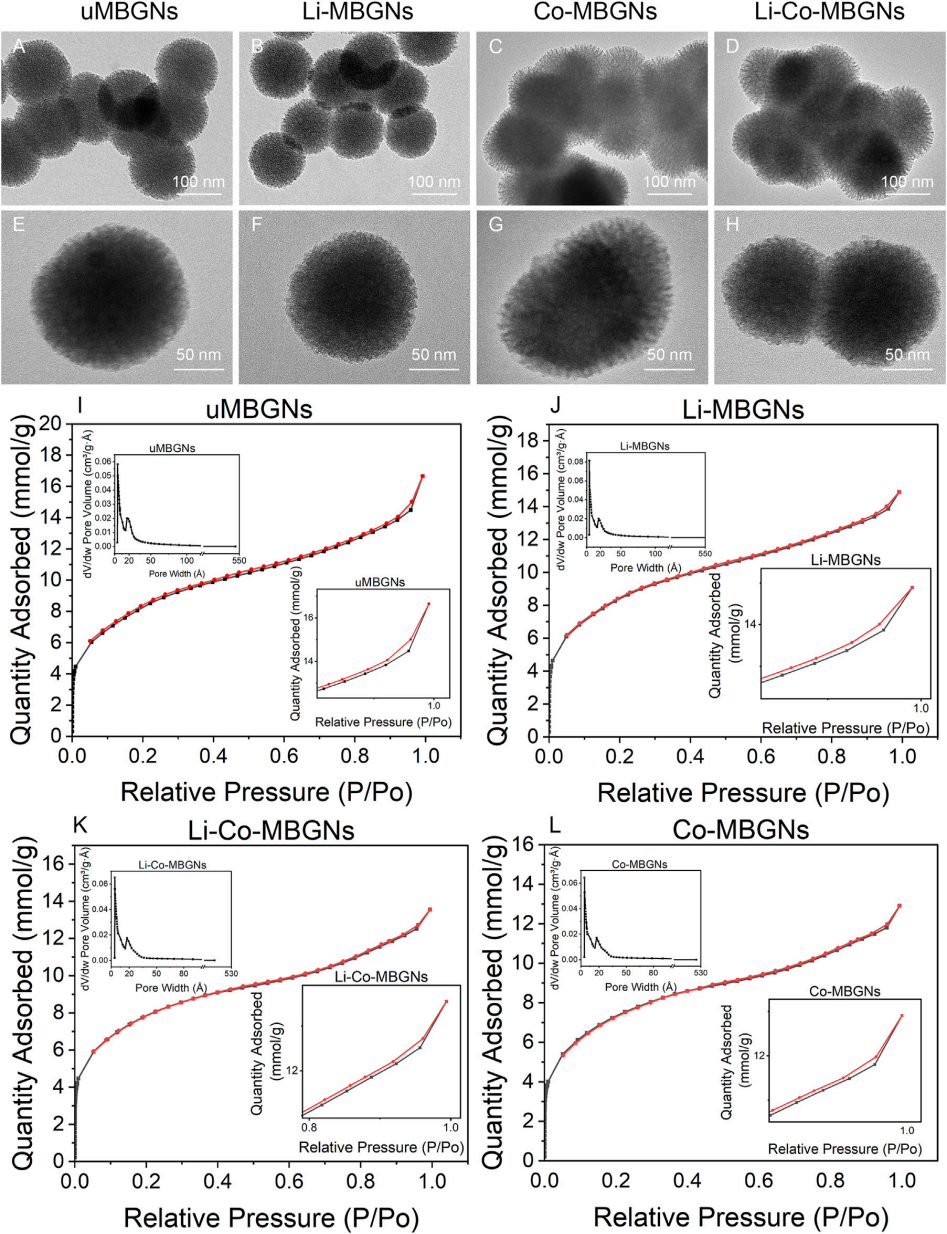
MBGN textures. **(A–H)** TEM images. **(I–L)** N^2^ adsorption-desorption isotherms and size distributions.

The hollow mesoporous structure of MBGNs provided a large, specific surface area to facilitate ion release and enhance bioactivity. The texture of MBGN was assessed using TEM and N_2_ adsorption-desorption. TEM (Figures 2A–H) showed that all samples had mesoporous structures. N_2_ adsorption-desorption (Figures 2I–L) showed that all MBGNs exhibited type IV isotherms associated with the mesoporous structure defined by the International Union of Pure and Applied Chemistry (IUPAC) (Brunauer et al., 1940; Zheng et al., 2020). The surface areas, mesopore volumes, and mesopore sizes are listed in Supplementary Table S6. The pore sizes ranged from 37 to 42 Å, which matched the definition of mesoporous structures and were consistent with previous studies that used CTAB as a template (Hu et al., 2014; Wang and Li, 2016). The average pore size of uMBGNs was slightly larger than that of the other three types. Although the surface area was affected by the pore size, shape, arrangement, and nanoparticle shape and size and varied as a function of composition, all samples maintained a high surface area (>600 m^2^/g). These results suggest that the incorporation of Li and/or Co into the MBGNs only slightly affected their surface area and structure.

### 3.2 MBGN bioactivity

It has been reported that MBGNs induce surface HA formation when immersed in SBF (KokuboT et al., 2006; Zheng et al., 2015). Therefore, we measured the effects of Li and Co doping on bioactivity using SEM, FTIR spectroscopy, and XRD.

**FIGURE 3:**
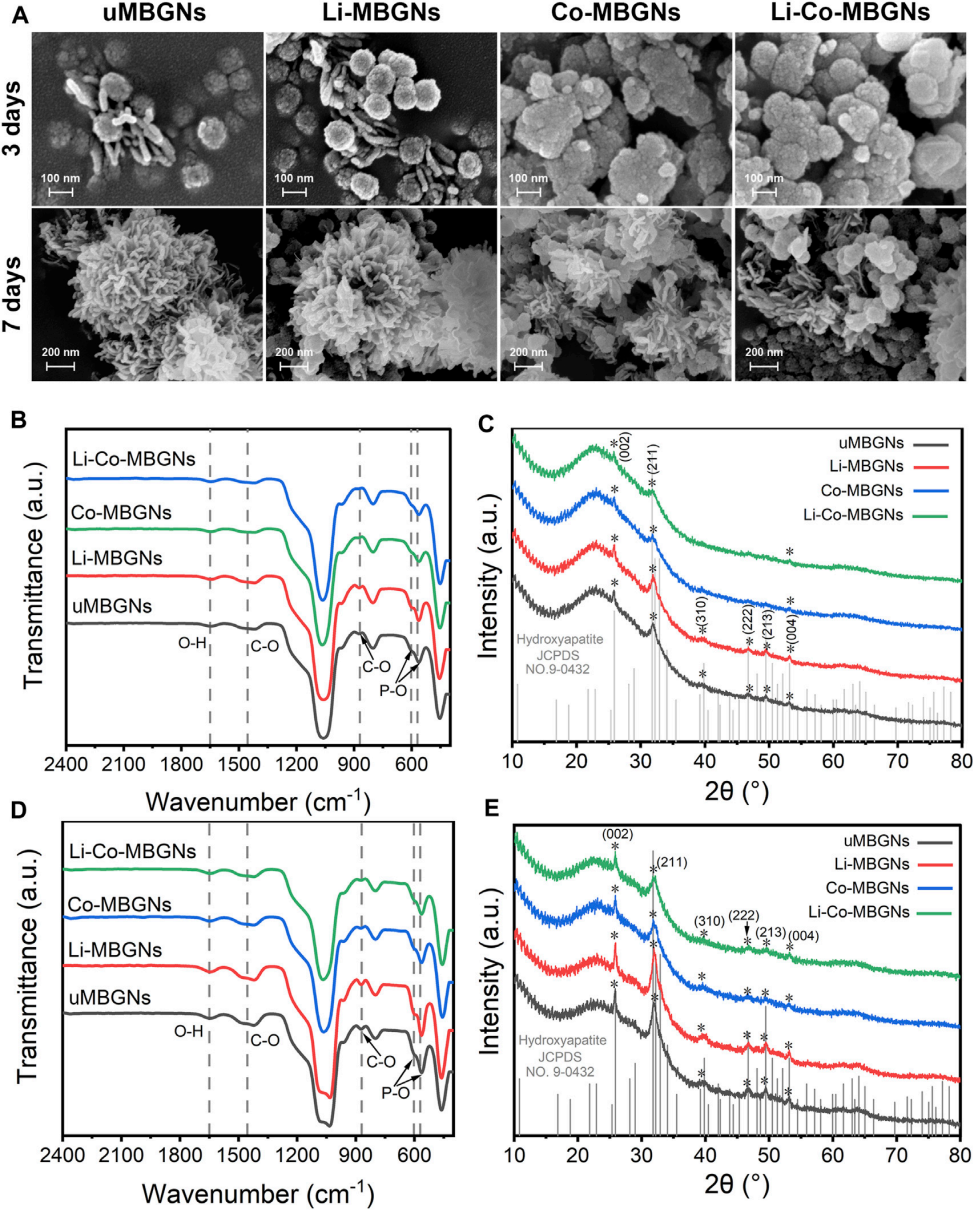
MBGN bioactivity. **(A)** SEM images after immersion in SBF for 3 and 7 days. FTIR spectra **(B)** and XRD patterns **(C)** after immersion in SBF for 3 days. FTIR spectra **(D)** and XRD patterns **(E)** after immersion in SBF for 7 days.

Using SEM (Figure 3A), globular agglomerates were observed in all samples after 3 days of immersion in SBF. However, only the uMBGNs and Li-MBGNs were typical needle-like HA crystals with a length of 100 nm and width of 10 nm present around the nanoparticles. After 7 d, needle-like crystals were observed in all types, forming cauliflower-like structures in the uMBGNs and Li-MBGNs. These results suggest that c-MBGNs and Li-MBGNs have significantly faster HA formation rates than Co-MBGNs and Li-Co-MBGNs.

FTIR spectroscopy was performed to characterize mineralization in the SBF. As shown in Figures 3B, D, bands at 570 cm^−1^ and 603 cm^−1^ corresponded to P–O bending, and bands at 1455 cm^−1^ and 870 cm^−1^ corresponded to C–O stretching, indicating surface HA formation (Moghanian et al., 2017). In contrast to c-MBGNs and Li-MBGNs, Co-MBGNs and Li-Co-MBGNs showed relatively lower intensities for these bands, which increased with longer immersion times for all four types.

XRD (Figures 3C, E) showed that the characteristic peaks assigned to the (002) and (211) planes could be identified at 25.9° and 31.8°, respectively, with increasing intensities as a function of the immersion time. The (310), (222), (213), and (004) planes were observed at 39.8°, 46.7°, 49.5°, and 53.1°, respectively. Although no obvious differences were observed between the Co-MBGNs and Li-Co-MBGNs, the peaks at 25.9° and 31.8° identified for the c-MBGNs and Li-MBGNs were stronger.

### 3.3 Ion release

**FIGURE 4:**
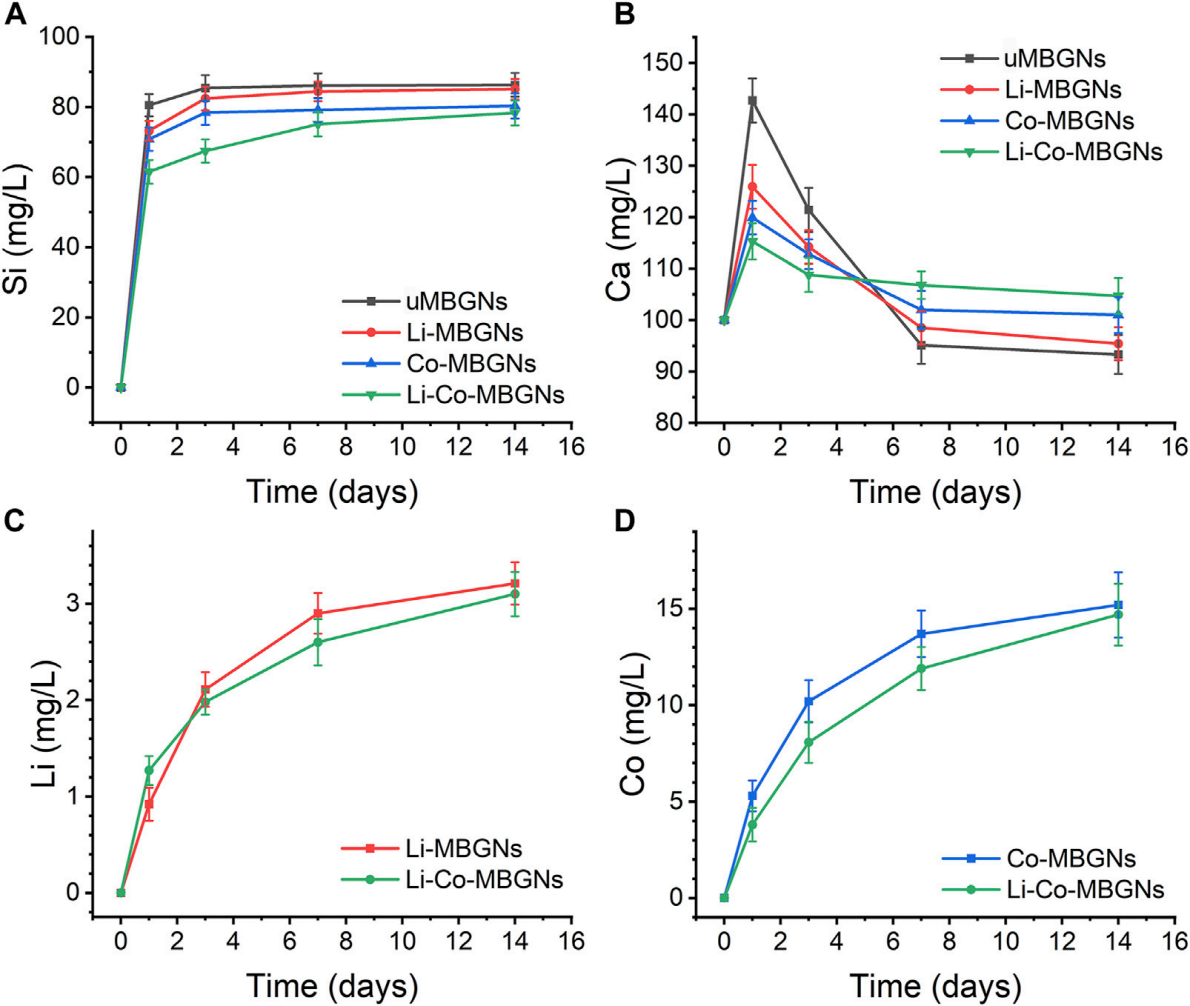
Ion release. **(A–D)** Release profiles of Si (SiO_4_^4-^) **(A)**, Ca (Ca^2+^) **(B)**, Li (li^+^) **(C)**, Co (Co^2+^) **(D)**, determined by optical emission spectroscopy with inductively coupled plasma (ICP-OES), after MBGN immersion in SBF for 1, 3, and 7 days. Data shown are mean ± SD (*n* = 3).

The release kinetics of multiple ions in SBF were evaluated using ICP-OES. As shown in Figure 4A, all groups exhibited an initial burst release of Si^4+^ within the first day, followed by a relatively stable and slow release from days 3–14. The amount of Si^4+^ released from uMBGNs was higher than that released from Li- and Co-doped BGNs, which is consistent with the data for Li-doped BGs reported by Khorami et al. (2011) and Co-doped BGs reported by El-Fiqi et al. (El-Fiqi and Kim, 2021). As shown in Figure 4B, all MBGNs displayed a rapid release of Ca^2+^ within 1 d, with uMBGNs releasing more than the other groups. This could be attributed to the fact that Li^+^ and Co^2+^, as dopants, enhance glass stability and partially replace Ca^2+^. Subsequently, Ca^2+^ release decreased gradually owing to its consumption during HA formation. Although Li and Co co-doping reduced the Ca^2+^ release, the desired level was still achieved with longer treatment times (Figures 4C, D). The release profiles of Co^2+^ and Li^+^ were similar, following a sustained pattern. Moreover, neither Li nor Co co-doping affected the Li^+^ and Co^2+^ release kinetics, endowing them with favorable biological properties. These results suggest that Li-Co-MBGNs have the potential for bone tissue engineering applications.

### 3.4 Antibacterial activity

**FIGURE 5:**
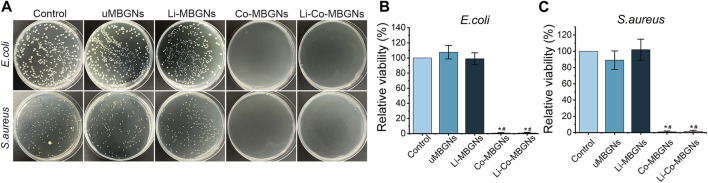
MBGN antibacterial activity. **(A)** Culture plates of *S. aureus* and *E. coli* after exposure to diverse samples. **(B, C)** Fractional survival of *E. coli* **(B)** and *S. aureus* **(C)** after 24-h culture. Data shown are mean ± SD (*n* = 3). *p < 0.05, significant with respect to control; #*p* < 0.05, significant with respect to uMBGNs.

*Escherichia coli* (*E. coli)* and *Staphylococcus aureus (S. aureus),* as representatives of Gram-negative and -positive bacteria, respectively, were chosen because they are common causes of bone infections. As shown in Figure 5, both *E. coli* and *S. aureus* grew well in the control, uMBGNs-, and Li-MBGN-treated conditions. In contrast, the growth of both species was markedly reduced when incubated with Co-MBGNs or Li-Co-MBGNs, suggesting that Co inhibited bacterial proliferation and that the incorporation of Li did not have antibacterial efficacy.

### 3.5 Biocompatibility

**FIGURE 6:**
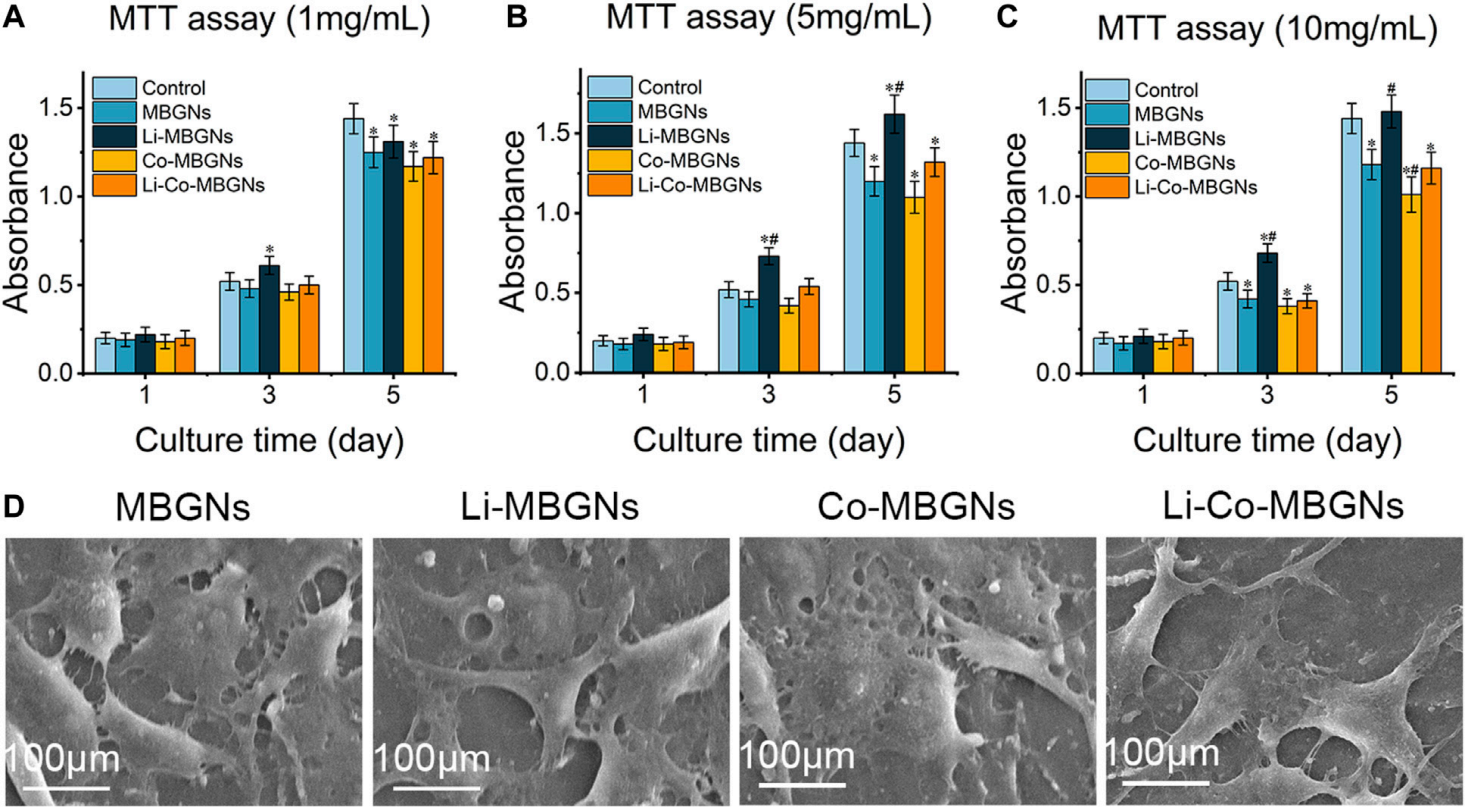
The MTT assay results of BMSCs cultured in α-MEM containing extracts of MBGNs at concentrations of 1 mg/mL **(A)**, 5 mg/mL **(B)** and 10 mg/mL **(C)**, respectively. **(D)** The images of the morphology of BMSCs attached to the different MBGN tablets. Data shown are mean ± SD (*n* = 3). * (*p* < 0.05) significant difference with respect to the control; # (*p* < 0.05) significant difference with respect to the uMBGNs.

To assess the effect of MBGN extracts on cell behavior, we performed 3-(4,5-dimethylthiazol-2-yl)-2,5-diphenyltetrazolium bromide (MTT), alkaline phosphatase (ALP), and cell adhesion assays on cultured BMSCs. In the MTT assay (Figures 6A–C), on day 1, no significant differences in cell proliferation were observed among the MBGN types at all concentrations of MBGN extracts. The cell viability of the uMBGNs was slightly lower than that of the controls, as the extract concentration increased, the inhibitory effect became more evident. In comparison to uMBGNs, the proliferation of Li-MBGNs was significantly increased, particularly at a concentration of 5 mg/mL. Notably, the proliferation rate of Li-MBGNs even exceeded that of the control group on days 3 and 5. On the contrary, Co-MBGNs exhibited a notable inhibition of proliferation. This inhibitory effect became increasingly evident on day 5, and as the concentration increased, the suppressive impact intensified. Intriguingly, the co-doping of Li and Co showed a compensatory effect, particularly mitigating the inhibitory impact of Co-MBGNs on the cell viability. Remarkably, at a concentration of 5 mg/mL, the cell viability even exceeded that of the uMBGNs group on days 3 and 5. ALP activity (Supplementary Figure S2) increased in a time-dependent manner for all MBGN types, with Li-Co-MBGNs at each time point being higher than that of MBGNs, demonstrating that the co-incorporation of Li and Co promotes functional BMSC differentiation.

As the attachment and expansion of cells on a material surface are regarded as important indicators of biocompatibility, we observed BMSCs on the surface of MBGN tablets (MBGNTs) using SEM to verify their adhesion and spreading (Figure 6D). After 3 days of incubation, the BMSCs adhered to the surface of the tablets and exhibited a well-spread morphology with numerous filopodia, indicating compatibility.

### 3.6 MBGN effects on osteogenic potential

**FIGURE 7:**
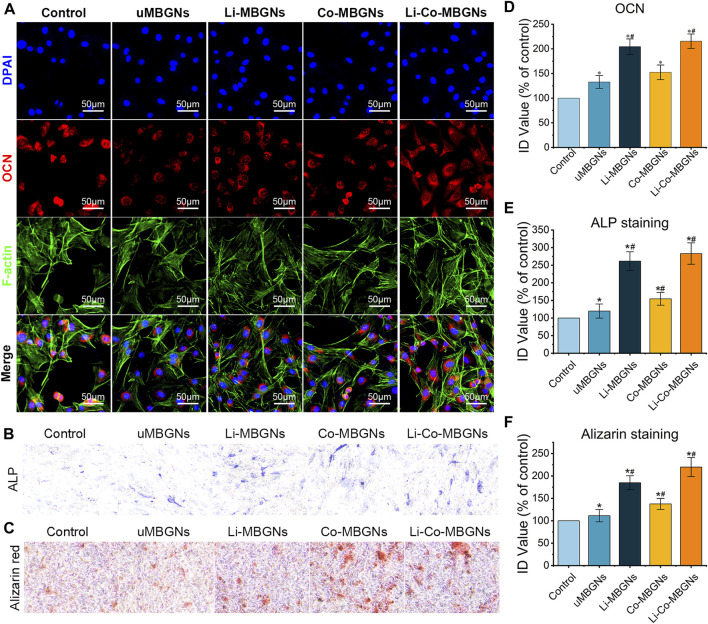
MBGN osteogenicity. Immunofluorescence staining for OCN **(A)**, ALP staining **(B)**, and Alizarin staining **(C)** of BMSCs cultured in α-MEM supplemented with MBGN extracts. **(D–F)** Integral density (ID) quantification of immunofluorescence **(D)**, ALP staining **(E)**, and Alizarin staining **(F)**. Data shown are mean ± SD (*n* = 3). *p < 0.05, significant with respect to control; #*p* < 0.05, significant with respect to uMBGNs.

Immunofluorescence was used to visualize osteocalcin (OCN) expression in the BMSCs exposed to MBGN extracts. As shown in Figures 7A, D, the red fluorescence intensity of uMBGNs and Co-MBGNs was slightly higher than that in control cells, whereas it markedly increased with Li-MBGNs and Li-Co-MBGNs, indicating that Li doping significantly enhanced osteogenic differentiation. ALP activity is a well-established marker of early osteogenesis, and *in vitro* mineralization is a key indicator of the late-stage osteogenic differentiation of BMSCs. ALP staining (Figures 7B,E) revealed that BMSCs exposed to Li-MBGN and Li-Co-MBGN extracts exhibited more ALP-positive areas and higher intensity; semi-quantitative analysis showed that the integral density (ID) values for Li-Co-MBGNs were approximately 2.5 times higher than those of the controls. Similarly, ARS staining demonstrated that Li doping (Li-MBGNs and Li-Co-MBGNs) enhanced BMSC mineralization (Figures 7C,F). The expression of genes encoding the osteogenesis markers ALP, OCN, OPN, and Runx2 increased over time in all types ((Supplementary Figure S3). Li-MBGN and Li-Co-MBGN substantially increased the expression of these genes compared with the other types on day 7, suggesting that incorporating Li promotes osteogenic differentiation.

### 3.7 MBGNs increase angiogenesis

**FIGURE 8:**
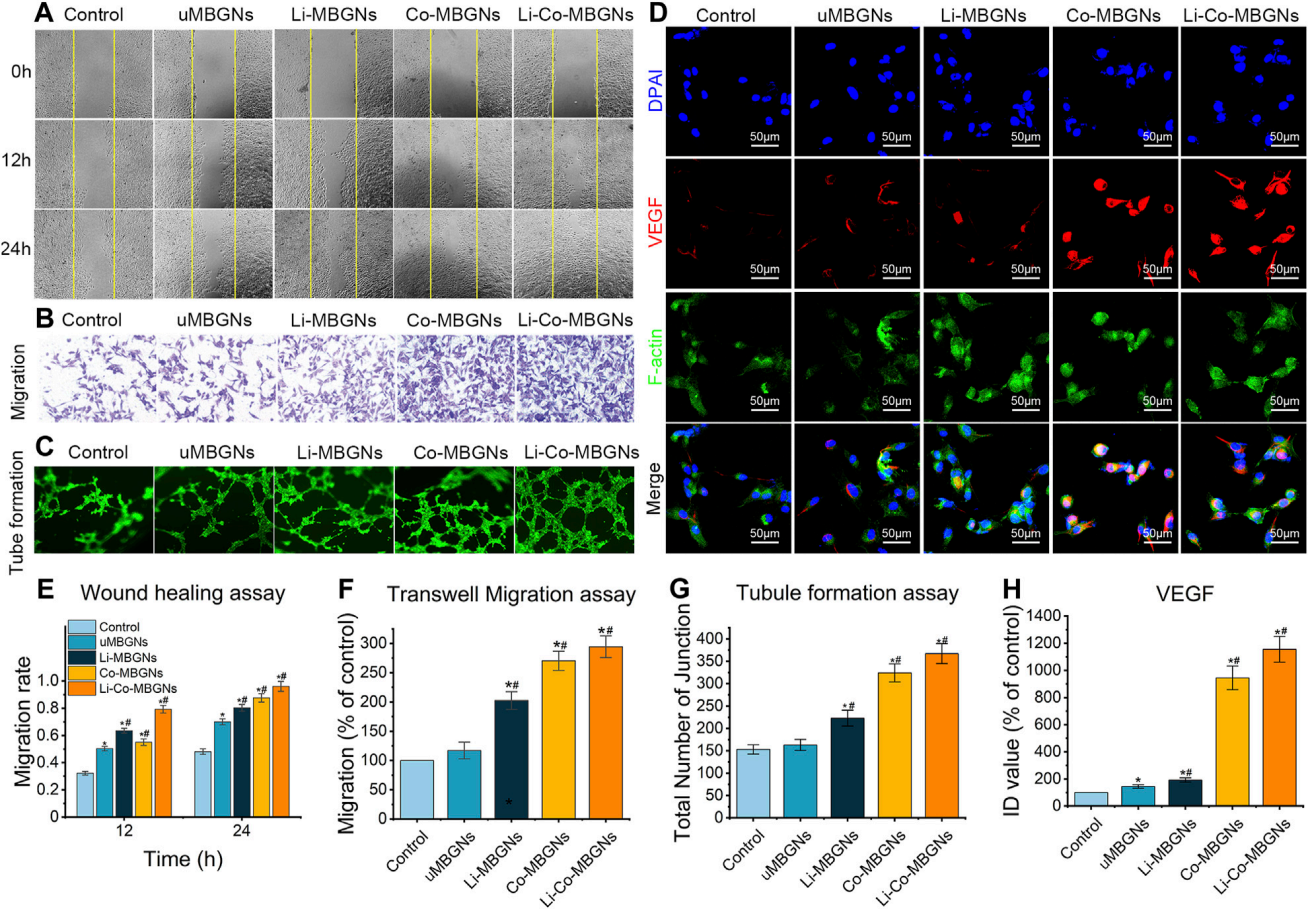
MBGNs promote angiogenic properties in wound healing **(A)**, Transwell migration **(B)**, tube formation **(C)** and VEGF immunofluorescence **(D)** assays on HUVECs cultured in α-MEM supplemented with MBGN extracts. **(E–H)** Quantification of Transwell I, tube formation **(F)**, immunofluorescence **(G)**, and wound-healing **(H)** assays. Data shown are mean ± SD (*n* = 3). *p < 0.05, significant with respect to control; #*p* < 0.05, significant with respect to uMBGN.s.

To examine the *in vitro* pro-angiogenic effects of MBGNs, we performed wound healing, Transwell, and tube formation assays using HUVECs (Liu et al., 2019). During wound healing (Figure 8A), scratches of the same initial width were made, and the HUVECs gradually migrated from the edge to the center of the wound area over time. After 24 h, the residual scratch width was widest in the control, followed by uMBGNs, Li-MBGNs, Co-MBGNs, and Li-Co-MBGNs, with migration ratios 1.5-, 1.7-, 1.8, and 2.0 times higher than that of the control, respectively (Figure 8E), indicating that Li and Co doping promoted HUVEC migration, with a more pronounced effect observed with their combination. Similar results were obtained in the Transwell assays (Figures 8B,F). More HUVECs migrated through the membrane with Li-MBGNs, Co-MBGNs, and Li-Co-MBGNs than with the control and uMBGNs. Tube formation assays (Figures 8C,G) showed that Co-MBGNs and Li-Co-MBGNs promoted more cell-cell junctions than the other three conditions, especially Li-Co-MBGNs.

VEGF expression (Figure 8D) was increased by Co-MBGNs and Li-Co-MBGNs over the other three conditions, with an ID value for Li-Co-MBGNs nearly six times higher than that for c-MBGNs (Figure 8H), suggesting that Co doping endowed MBGNs with a significant angiogenic capacity. Finally, qRT-PCR was conducted to measure the expression of genes encoding the angiogenesis markers HIF-1α, VEGF, and KDR after incubation with MBGNs. As shown in (Supplementary Figure S4, the mRNA levels of KDR and VEGF with Co-MBGNs and Li-Co-MBGNs were significantly higher than those in the other conditions on days 3 and 7; however, there was no significant difference in HIF-1α between the control and experimental groups. In conclusion, Li incorporation slightly increased the expression of angiogenesis markers with or without Co incorporation.

## 4 Discussion

Bone regeneration is a complex process involving multifaceted biological events, including inflammation, osteogenesis, angiogenesis, and peripheral nerve regeneration (Zhu G. et al., 2021). A promising strategy for bone regeneration is to modulate these events through multiple bioactive components such as genes, drugs, and ions, which elicit specific cellular responses and improve biomaterial performance. However, optimal selection and delivery of these components for bone tissue engineering remain challenging. These effects vary depending on the cell type and stage of bone healing. Flexible and effective approaches are required to spatiotemporally control these events. Bioactive glass (BG) has gained increasing attention because of its biocompatibility, biodegradability, bioactivity, and osteoconductivity. However, their osteoinductive and angiogenic properties are inadequate to fulfill the requirements of bone regeneration. In this study, multifunctional biomaterials were developed by doping MBGNs with Li and Co, based on their advantageous biological properties.

The differences in composition lead to differences in silicate network structure, leading to variations in the biological activity, and ion release of MBGNs (Brauer, 2015). The ^29^Si MAS NMR results showed that the incorporation of Li and Co significantly decreased the degree of polymerization and network connectivity of the silicate network of the MBGNs. However, the FTIR spectra showed no significant differences among the four types of MBGNs, which was in agreement with previous studies (Kargozar et al., 2016; El-Fiqi and Kim, 2021; Simila and Boccaccini, 2023). It is possible that Ca^2+^, Li^+^, and part of the Co^2+^ exist in the silicate network as modifiers, which are ionically linked to nonbridging oxygen atoms, and it is not easy for FTIR to detect the ionic bonds. Some Co^2+^ exists as a network constituent, but it is difficult to detect because of its low content. In addition, the absorption bands of the Ca-O, Li-O, and Co-O stretching vibrations, mainly ranging from 450 to 660 cm^-1^, were readily obscured by Si-O-Si in the silicate network.

In this study, Li-MBGNs were smaller than uMBGNs, consistent with the results of a previous study (Simila and Boccaccini, 2023); however, explanations for this were not provided. The incorporation of Co^2+^ results in nanoparticle agglomeration. Given that Co^2+^ has a smaller radius and higher field strength than Ca^2+^, it is easier to adsorb onto the surface of MBGNs. We assumed that the substitution of Ca^2+^ by Co^2+^ increased the metal cation content in the silicate network, which in turn counteracted the negative surface charge of the MBGNs and increased their tendency to agglomerate, as evidenced by the ζ-potential results. However, the agglomerations were ultrasonically dispersed to the nanoscale, as demonstrated by the SEM images and DLS results. This has little effect on their application as inorganic nanoparticle additives in biological composite materials.

The ion release and bioactivity of MBGNs are associated with component and silicate network connectivity (Brauer, 2015). The incorporation of Co^2+^ decreased the silicate network connectivity, as evidenced by ^29^Si MAS NMR, which has a positive effect on ion release and HA formation. In this study, the incorporation of Li^+^ and Co^2+^ slowed Ca^2+^ release, and Co^2+^ delayed HA formation on the surface of the MBGNs. This slowness and delay have also been observed in previous studies (Azevedo et al., 2010; Kargozar et al., 2016; Barrioni et al., 2018; El-Fiqi and Kim, 2021; Simila and Boccaccini, 2023). Brauer et al. explained that the substitution of Ca^2+^ by Co^2+^ led to a decrease in Ca^2+^ content, resulting in lower Ca^2+^ release and a delay in HA formation (Azevedo et al., 2010). Additionally, given that Co^2+^ has a smaller radius than Ca^2+^, the incorporation of Co^2+^ compacted the silicate network compared to pure MBGNs, slowing the penetration of water molecules, and subsequently resulting in decreased Ca^2+^ release and delayed HA formation.

Unlike the cell assay, the anti-bacterial assay was performed using the spread plate technique, in which bacteria were cultured on solid media. This has a greater resemblance to the application scenario of MBGNs. Therefore, an anti-bacterial assay was conducted using a direct contact method. These findings indicate that the anti-bacterial effects of Co-MBGNs and Li-Co-MBGNs were primarily attributed to the presence of Co^2+^ ions. Previous studies have shown that the presence of Co in silicate microspheres, montmorillonite, hydroxyapatite, and oxide ceramics results in excellent antibacterial activity (Talebian and Zare, 2014; Boldbaatar et al., 2019; Shi et al., 2019; Bhattacharjee et al., 2020; Yang S. et al., 2022), which agrees with our results. According to Barras et al., Co exerts antibacterial effects primarily by interfering with iron homeostasis via its interaction with Fe-S clusters, which are essential cofactors for DNA repair and respiration (Barras and Fontecave, 2011; Raja et al., 2023).

Biocompatibility is an indispensable prerequisite for the successful clinical application of biomaterials, and its application scenarios determine evaluation methods. MBGNs primarily serve as versatile building blocks and are commonly incorporated into composite biomaterials such as 3D printed scaffolds and composite hydrogels (Vallet-Regi and Salinas, 2021). In these cases, direct contact between MBGNs and cells is difficult, and the biological effects of MBGNs are predominantly attributed to the dissolution products and leachates they generate. Consequently, similar to previous research (Yang X. et al., 2022), an extraction-based approach is an appropriate choice for the biological evaluation of MBGNs. In addition, MBGNs are also used in orthopedic implant coatings, where direct cell adhesion occurs on their surfaces. The surface structure of MBGNs can also affect cell behavior; therefore, cell adhesion and morphology were also evaluated on the surfaces of the MBGN tablets.

Compared to the control, the uMBGN extracts exhibited a reduction in cell viability, consistent with findings from previous investigations (Bejarano et al., 2015; Westhauser et al., 2021b; Moghanian et al., 2021). This phenomenon can be attributed to the elevated pH levels in the medium resulting from the release of cations derived from MBGNs (Bejarano et al., 2015). Notably, the introduction of Li^+^ significantly enhanced cell viability compared to uMBGNs, as demonstrated by previous studies (Khorami et al., 2011; Liu et al., 2019). This effect can be elucidated by the activation of the Wnt canonical signaling pathway mediated by Li^+^ (Liu et al., 2019). The incorporation of Co^2+^ exhibited a reduction in cell proliferation, particularly at a high concentration. Similar cytotoxicity, in a dose-dependent manner, could been found in previous studies (Wu et al., 2012; Solanki et al., 2021). In addition, Co^2+^ exhibited cytotoxicity also in a time-dependent manner (Fleury et al., 2006; Ahamed et al., 2016). Those were considered to be associated with the reactive oxygen species (ROS), apoptosis and DNA damage induced by Co^2+^ (Song et al., 2012; Alarifi et al., 2013; Ahamed et al., 2016). Intriguingly, the augmenting influence of Li^+^ was observed to offset the diminishing effect caused by Co^2+^ in the cell viability to some extent, as evidenced by the higher cell viability observed in Li-Co-MBGNs compared to Co-MBGNs.

Previous studies demonstrated that Li in the cytoplasm inhibits glycogen synthase kinase-3β (GSK-3β), which phosphorylates β-catenin and promotes its degradation via ubiquitination (Han et al., 2012; Han et al., 2014). With increased cytoplasmic content, β-catenin moves to the nucleus and activates a series of proliferation- and osteogenesis-related genes (Yuan et al., 2019). Based on these phenomena, lithium has been widely used as an osteogenic stimulant in biological tissue engineering and has been incorporated into multiple biomaterials for the regeneration and repair of bone defects, including bioactive scaffolds (Han et al., 2012; Wu et al., 2014), β-tricalcium phosphate (Yuan et al., 2019), and bioceramic scaffolds (Shi et al., 2015). We observed that the incorporation of Li into MBGNs significantly enhanced the osteogenic differentiation of BMSCs and upregulated the expression of osteogenesis-related genes, which is in agreement with the results of previous studies. Although there was no significant improvement in osteogenesis mediated by Co-MBGNs, it should be noted that we observed a potential for it to enhance osteogenesis in the form of a slight increase in the expression of osteogenesis markers.

Cobalt can induce a cytoplasmic hypoxic cascade, and its incorporation into biomaterials can improve their angiogenic potential. A previous study revealed that cobalt stabilizes HIF-1α in the cytoplasm by inhibiting its interaction with the von Hippel-Lindau protein, promoting the rapid degradation of HIF-1α via ubiquitination (Yuan et al., 2003). Following its stabilization and concentration in the cytoplasm, HIF-1α is shuttled into the nucleus, combines with HIF-1β to form HIF-1, and activates the transcription of genes related to angiogenesis and tissue regeneration (Yuan et al., 2003). This study showed that Co-MBGNs and Li-Co-MBGNs induced increased expression of angiogenesis-related genes, such as VEGF and KDR, compared to MBGNs, indicating that Co is an attractive dopant, which is consistent with many studies in the literature (Wu et al., 2012; Laia et al., 2021; Yang S. et al., 2022). Moreover, a large body of literature suggests that Si and Li ions released from biomaterials promote angiogenesis (Hedgepeth et al., 1997; Liu et al., 2019). Lidoping enhanced VEGF expression; however, this enhancement was not significantly greater than that induced by Co doping.

This study has some limitations that should be acknowledged. First, we measured the ion release by Li-Co-MBGNs, but we did not precisely quantify their composition, which may have affected the accuracy and reproducibility of our results. Second, we only performed *in vitro* osteogenic and angiogenic experiments without *in vivo* experiments to validate our findings. As a result, the biocompatibility and bioactivity of Li-Co-MBGNs in animal models and humans remain unknown. Thirdly, we did not evaluate the potential of Li-Co-MBGNs for drug delivery by performing drug loading and release experiments. Future studies should address these limitations to better understand the mechanisms and applications of Li-Co-MBGNs in bone tissue engineering.

## 5 Conclusion

In this study, Li-Co-MBGNs were synthesized using a sol-gel method with CTAB as a template agent based on the SiO_2_-CaO-P_2_O_5_ system. They exhibited irregular spherical shapes with a mean size of 121 ± 19 nm. The nanoparticles tended to aggregate, but the aggregates were dispersed into nanoscale pieces. The incorporation of Li and Co did not influence the amorphous structure and textural properties of the BGNs but resulted in a mesoporous structure and a high specific surface area. The co-incorporation of Li and Co delayed the formation of HA and did not negatively affect the sustained release kinetics of the therapeutic ions. The addition of Co endowed the MBGNs with significant antibacterial properties against *S. aureus* and *E. coli*. At optimal concentrations, Li-Co-MBGNs did not show obvious cytotoxicity towards BMSCs and HUVECs and presented excellent pro-osteogenic differentiation ability in BMSCs and pro-angiogenesis ability in HUVECs. Future research should focus on the role of Li-Co-MBGNs as nanofillers in composite biomaterials such as 3D printing inks and injectable hydrogels for applications in bone tissue engineering. Overall, Li-Co-MBGNs have great potential as biomaterials in bone tissue engineering.

## Data Availability

The datasets presented in this study can be found in online repositories. The names of the repository/repositories and accession number(s) can be found in the article/Supplementary Material.
