# Supplementary material for: Lithium and cobalt co-doped mesoporous bioactive glass nanoparticles promote osteogenesis and angiogenesis in bone regeneration
Source: Front Bioeng Biotechnol. 2024 Jan 4;11:1288393. doi: 10.3389/fbioe.2023.1288393 (PMC10794388; doi:10.3389/fbioe.2023.1288393)
Supplement: Supplementary file 1 [file DataSheet1.DOCX]

Supplementary Material

Fabrication of Lithium and cobalt co-doped mesoporous bioactive glass nanoparticles for bone regeneration by promoting osteogenesis and angiogenesis

Xin Zhang, Kai Nan, Yuankai Zhang, Keke Song, Zilong Geng, Donglong Shang, Lihong Fan

*** Correspondence:** Lihong Fan: drfan2140@xjtu.edu.cn

1. **Materials and** **Methods**

**1.1 Antibacterial experiment in vitro**

To assess the antibacterial performance of different MBGNs, the spread plate method was adopted, as described in a previous study(10). First, Escherichia coli (*E. coli*) and Staphylococcus aureus *(S. aureus)* were amplified in Luria Bertani (LB) medium at 37 °C overnight and then diluted to 2 × 10^5^ colony-forming units per mL (CFU/mL) with PBS. The bacterial cells were collected and maintained at 37 °C under agitation. Different MBGNs (50 mg) were transferred into the mixed solution composed of 47.5 mL of the stock solution at pH 7.4 and 2.5 mL of nutrient broth, and the mixture was incubated at 37 °C under agitation overnight. Next, 100 μL of the mixture was inoculated onto LB agar plates and incubated at 37 °C for another 24 h. The number of colony-forming units was counted to assess antibacterial activity. The tests were performed in triplicates.

**1.2 Cell proliferation assay**

To assess the effect of different types of MBGNs on cell proliferation, the 3-(4,5-dimeth-ylthiazol-2-yl)-2,5-diphenyl tetrazolium bromide MTT assay was performed using BMSCs. First, hBMSCs cells were seeded in 96-well plates at a density of 1×10^4^ cells per well and cultured in α-MEM at 37 °C under 5% CO_2_ conditions. After incubation for 24 h, the culture medium was replaced with a medium supplemented with the extracts. After another 24 h incubation, MTT solution was added to each well and incubated at 37 °C under 5% CO_2_ conditions for 4 h to form formazan crystals. The absorbance of the resultant solution was measured at 495 nm using a plate reader. The experiments were performed in triplicate.

**1.3 Alizarin red staining assay (ARS)**

To determine the mineralization ability of MBGNs, an alizarin red staining assay (ARS) was applied. First, BMSCs were seeded in 6-well plates at a density of 1 × 10^5^ cells per well. The cells were cultured in the α-MEM containing osteogenic supplements and the extracts of MBGNs in 5% CO_2_ at 37 °C for 15 days, and the medium was changed every 3 days. After removal of the medium, the cells were washed with PBS three times and then fixed with 4% paraformaldehyde for 30 minutes. Subsequently, the cells were stained with 40 mM Alizarin Red S solution (pH 4.2) for 10 min at 37 °C. The cells were then washed with distilled water three times. The mineralized structure formed was observed using a high-contrast inverted microscope. The experiments were performed in triplicate

**1.4 Alkaline phosphatase (ALP) staining and activity**

ALP assays were conducted as previously described (6, 11). The hBMSCs were seeded in 24-well culture plates with α-MEM containing osteogenic supplements and extracts of MBGNs at a concentration of 5 mg/mL (1 × 10^5^ cells per well) and then incubated under 5% CO_2_ conditions at 37 °C for 7 days. At the predetermined time, ALP staining was conducted as previously reported(11). Briefly, the cells were washed twice with PBS and fixed with 4% paraformaldehyde for 15 min. After washing twice with PBS, the staining work solution, prepared according to the instructions of the ALP staining kit (Beyotime, China), was added into the wells for 30 min away from light before taking photos. The supernatant in each well was removed, and ALP activity was measured(6). The cells were washed with PBS three times, and then 0.5 mL of 0.02% Triton X-100 was added into the wells to dissolve the cells. The solution was transferred into a 1.5 mL tube and sonicated. Next, the samples were centrifuged at 14,000 rpm for 15 min at 4 °C. The supernatant was transferred to fresh 1.5 mL tubes, and 100 μL of 1 mol/L Tris-HCl, 20 μL of 5 mmol/L MgCl_2_, and 20 μL 5 mmol/L p-nitrophenyl phosphate were added. After 30 min incubation at 37 °C, the reaction was stopped by adding 50 μL of 1 N NaOH. Using p-nitrophenol as a standard, the optical density was measured at 410 nm using a spectrophotometer. Moreover, the protein content was determined by using the bicinchoninic acid (BCA) assay kit. ALP activity was obtained using the following formula:

$$\boldsymbol{ALP activity}=\frac{\boldsymbol{OD}_{\boldsymbol{n}}\boldsymbol{-}\boldsymbol{OD}_{\boldsymbol{0}}}{\boldsymbol{T\times}\boldsymbol{P}_{\boldsymbol{t}}}$$

where ${OD}_{n}$ represents the OD value at the predetermined time, ${OD}_{0}$ represents the initial OD value, T represents the reaction time, $P_{t}$ represents the total protein quantity.

**1.5 Tubule formation in vitro**

Tubule formation was assayed to evaluate the effect of the MBGN extracts on HUVEC angiogenesis, Matrigel was thawed at 4 °C overnight and added to a 24-well plate (250 μL per well), followed by incubation at 37 °C for 60 min to solidify. Cells (1× 10^5^ per well) were seeded on a Matrigel matrix and cultured in a complete medium supplemented with MBGN extracts. After 12 h, cells were identified using immunofluorescence staining, The number of tubes and branch points were counted using ImageJ software. The experiments were performed in triplicate

**1.6 *W*ound healing and Transwell assays**

For the wound healing assay, HUVECs were seeded in a 6-well plate at a density of 1 × 10^6^ cells per well and incubated in 5% CO_2_ at 37 °C overnight. When the cells adhered to the plates, scratches were created using 200 μL pipette tips on the monolayer of HUVECs. The culture medium was then replaced with media containing MBGN extracts, and HUVECs were cultured at different times. Images were taken using an inverted phase microscope at 0, 12, and 24 h. The migration rate (%) was obtained using the following formula:

$$\boldsymbol{Migration rate}\boldsymbol{(\%)=}\frac{\boldsymbol{A}_{\boldsymbol{0}}\boldsymbol{-An}}{\boldsymbol{A}_{\boldsymbol{0}}}\boldsymbol{\times100\%}$$

where *A_0_* represents the initial scratch area, and *A* represents the residual area at different time points.

Transwell assays were performed using 24-well Transwell cell culture inserts. The upper chambers were loaded with serum-free medium, and the HUVECs with a density of 4 × 10^4^ cells per well were seeded. The lower chamber was loaded with conditioned medium containing 10% FBS and MBGN extracts. After incubation at 37 °C in 5% CO_2_ for 24 h, the inserts were taken out of the upper chambers and washed with PBS, and the cells were fixed with 4% paraformaldehyde and stained with 0.1% crystal violet. The non-invading cells on top of the inserts were gently removed using a cotton swab. Images were captured using a fluorescence microscope. The migrated cells on each insert were counted in six randomly selected high-power fields (under a 20× objective lens) and quantified using the Image J software. All experiments were performed in triplicate.

**1.7 Quantitative real-time polymerase chain reaction (****qRT-PCR)**

To detect the expression of osteogenic-related markers, including ALP, OCN, OPN and RUNX-2 on days 1, 3 and 7, and the angiogenic-related genes of HIF-1α, VEGF, and KDR on days 3 and 7, quantitative real-time polymerase chain reaction (qRT-PCR) analysis was performed as previously described(13). The hBMSCs and HUVECs were cultured in the α-MEM medium containing MBGN extracts, and incubated in 5% CO_2_ at 37 °C for 3 and 7 days, respectively. Total RNA was extracted and quantified using Trizol Reagent® according to the manufacturer's instructions. Complementary DNA was synthesized from 1 μg of total RNA using the Reverse Transcription Kit PLUS (EZ Bioscience) as described by the manufacturer's instructions. Then, real-time PCR was performed in a final volume of 10 μL containing 1μL of cDNA, 1μL of each primer, 1μL of RNase-free water, and 5μL of SYBR Green fluorescent real-time PCR kit (Invitrogen, USA), and the reaction was performed using ABI 7900 HT Sequence Detection System. Glyceraldehyde phosphate dehydrogenase (GAPDH) was used as an internal control in hBMSCs and HUVECs, and the transcription level of each gene was normalized to GAPDH. The difference between the threshold cycle (Ct) of GAPDH and the target gene was calculated to obtain the Δ threshold cycle (ΔCt) value. The relative fold change in gene expression between the experimental and blank control groups was determined using the formula 2^−ΔΔCt^. The specific primer sequence for each gene was designed using Primer Premier software (Primer Premier 6.0) (6), Primer-BLAST program (http://www.ncbi.nlm.nih.gov/tools/primer-blast/), or following previous studies(12, 14, 15). Primer specificity was tested using Primer-BLAST. The primers used in this study are provided in Tables S1 and S2. The experiments were performed in triplicate.

# Supplementary Figures and Tables

## Supplementary Figures


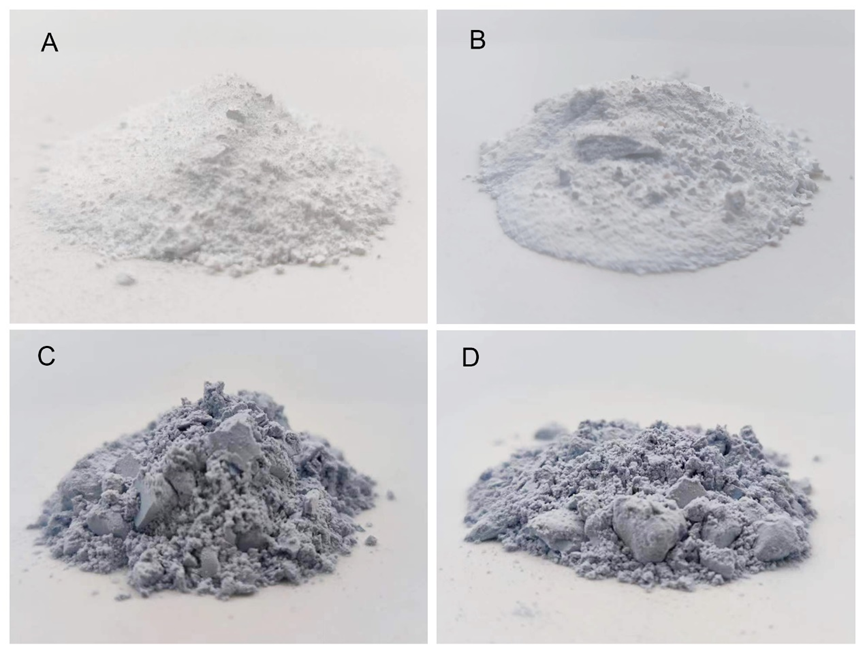


**Figure S1** The images of the four MBGN powders. (A) uMBGNs, (B) Li-MBGNs, (C) Co-MBGNs, and (D) Li-Co-MBGNs.


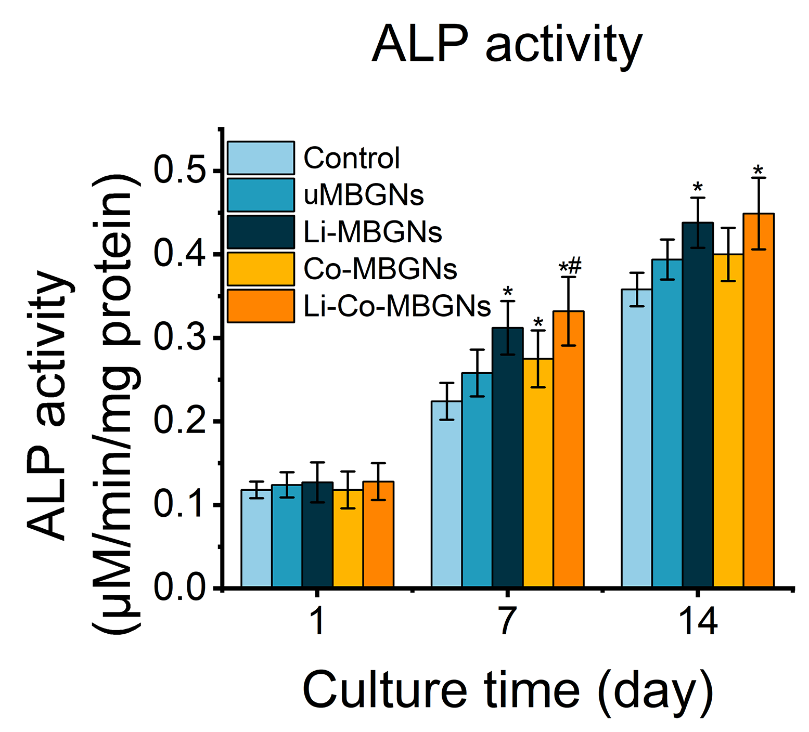


Figure S2 The ALP activity result of BMSCs cultured in α-MEM supplemented with extracts of different MBGNs at a concentration of 5 mg/ml. * (P<0.05) significant difference with respect to the control; # (P<0.05) significant difference with respect to the uMBGNs.


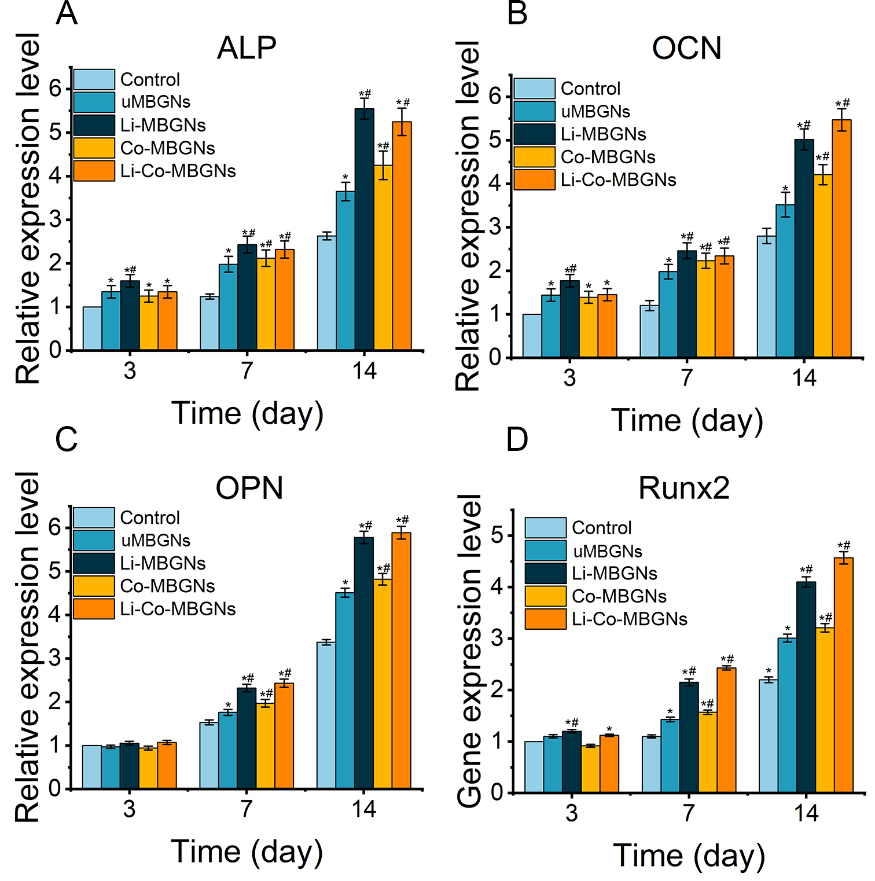


**Figure S3** The qRT-PCR analysis of osteogenesis-related genes ALP (A), OCN (B), OPN (C), and Runx2 (D), respectively. * (P<0.05) significant difference respect to the control; # (P<0.05) significant difference respect to the uMBGNs.


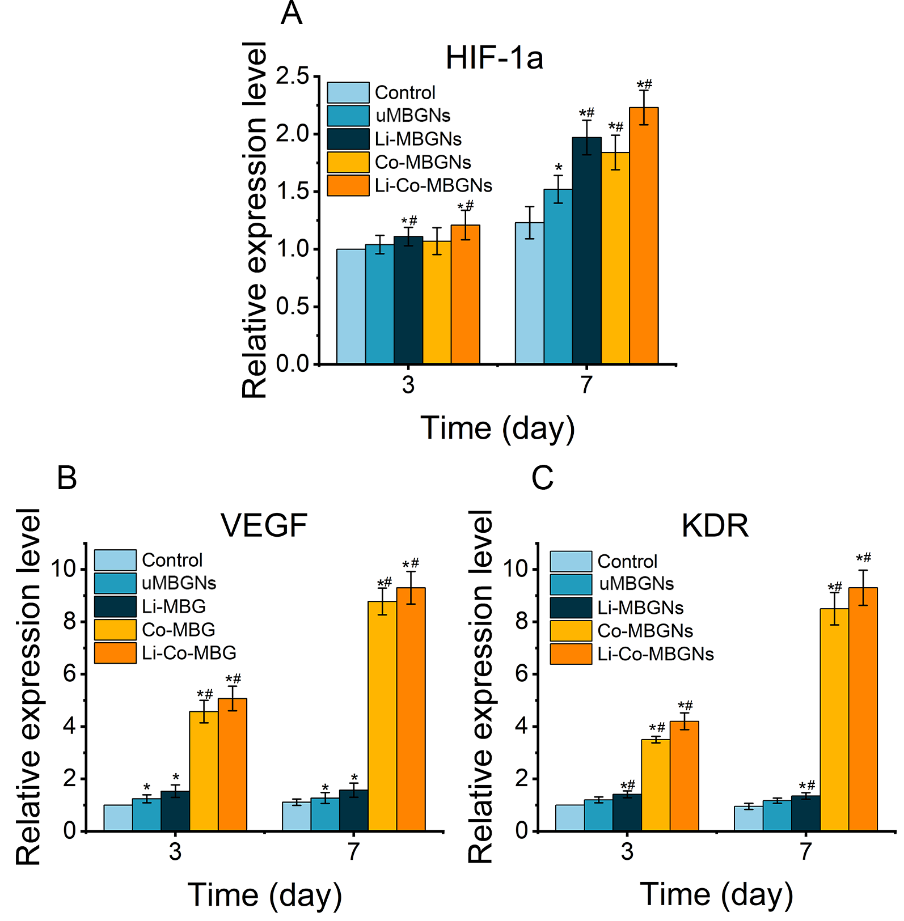


Figure S4 The qRT-PCR analysis of angiogenesis-related genes HIF-1α(A), VEGF (B) and KDR (C). * (P<0.05) significant difference with respect to the control; # (P<0.05) significant difference with respect to the uMBGNs.

## Supplementary Tables

Table S1 Nominal composition of the synthesized MBGNs (mol%)

|  | Si | P_2_ | Ca | Li_2_ | Co |
| --- | --- | --- | --- | --- | --- |
| uMGBNs | 70 | 4 | 26 | 0 | 0 |
| Li-MBGNs | 70 | 4 | 21 | 5 | 0 |
| Co-MBGNs | 70 | 4 | 24 | 0 | 2 |
| Li-Co-MBGNs | 70 | 4 | 19 | 5 | 2 |

Table S2 Primers for HUVECs

| Gene | Primers (F=forwards; R=reverse) | Accession numbers | Product size (bp) |
| --- | --- | --- | --- |
| *VEGF*(12) | F: 5'CTACCTCCACCATGCCAAGT3' | NM_001025366.3  NM_001025368.3  NM_001204385.2  NM_001025370.3  NM_001171622.2  NM_003376.6  NM_001033756.3  NM_001025369.3  NM_001025367.3  NM_001317010.1 | 187  187  187  187  187  187  187  187  187  187 |
|  | R: 5'CACACAGGATGGCTTGAAGA3' |  |  |
| *KDR*(12) | F: 5'GTGATCGGAAATGACACTGGAG3' | NM_002253.4 | 124 |
|  | R: 5'CATGTTGGTCACTAACAGAAGCA3' |  |  |
| *HIF-1α*(12) | F: 5'ATCCATGTGACCATGAGGAAATG3' | NM_001243084.2  NM_181054.3  NM_001530.4 | 124  124  124 |
|  | R: 5'CGGCTAGTTAGGGTACACTT3' |  |  |
| *β-actin*(14) | F: 5'GGGAAATCGTGCGTGACATTAAGG3' | NM_001101.5 | 185 |
|  | R: 5' CAGGAAGGAAGGCTGGAAGAGTG 3' |  |  |

Table S3 Primers for hBMSCs

| Gene | Primers (F=forwards; R=reverse) | Accession numbers | Product size (bp) |
| --- | --- | --- | --- |
| *ALP* | F: 5' CACGTCGATTGCATCTCTGG 3' | NM_000478.6  NM_001369803.2  NM_001369805.2  NM_001369804.2 | 141  141  141  141 |
|  | R: 5' GCCAGTACTTGGGGTCTTTC 3' |  |  |
| *OCN*(15) | F: 5' GCAAAGGTGCAGCCTTTGTG 3' | NM_199173.6  NM_001199662.1  NM_001199664.1  NM_001199663.1  NM_001199661.1 | 86  86  86  86  86 |
|  | R: 5' GGCTCCCAGCCATTGATACAG3' |  |  |
| *OPN*(15) | F: 5' TCACCTGTGCCATACCAGTTAA 3' | NM_000582.3  NM_001251830.2  NM_001040058.2 | 112  301  112 |
|  | R: 5' TGAGATGGGTCAGGGTTTAGC3' |  |  |
| *RunX2* | F: 5'CAGCAGCACTCCATATCTC3'  R: 5'GTCAGCGTCAACACCATC3' | NM_001015051.4  NM_001278478.2  NM_001369405.1  NM_001024630.4 | 175  175  175  175 |
| *GAPDH*(15) | F: 5′ TCAGCAATGCCTCCTGCAC3′ | NM_001357943.2  NM_001256799.3  NM_001289745.3  NM_001289746.2  NM_002046.7 | 117  117  117  117  117 |
|  | R: 5′ TCTGGGTGGCAGTGATGGC3′ |  |  |

**Table S4 Mean MBGN sizes measured by SEM, DLS size, and ζ-potential**

| MBGNs | Average size (nm) | DLS size (nm) | ζ-potential (mV) (pH=7.4) |
| --- | --- | --- | --- |
| uMBGNs | 166.2±14.8 | 269.0±0.7 | -31.5±0.5 |
| Li-MBGNs | 127.2±11.3 | 203.9±1.3 | -29.2±0.3 |
| Co-MBGNs | 141.7±21.3 | 591.5±8.6 | -24.2±0.7 |
| Li-Co-MBGNs | 121.3±19.4 | 454.1±7.5 | -23.8±0.8 |

**Table S5 ^29^Si-NMR parameters of different MBGNs**

|  | **Q4** | | **Q3** | | **Q2** | |  |
| --- | --- | --- | --- | --- | --- | --- | --- |
| **Sample** | **Peak position** | **Integral** | **Peak position** | **Integral** | **Peak position** | **Integral** | ***Dc*** |
|  | **(ppm)** | **(%)** | **(ppm)** | **(%)** | **(ppm)** | **(%)** |  |
| uMBGNs | -110.9 | 74.8 | -102.0 | 25.2 |  |  | 3.75 |
| Li-MBGNs | -110.7 | 73.2 | -101.1 | 26.8 |  |  | 3.73 |
| Co-MBGNs | -110.4 | 71.9 | -100.6 | 21.8 | -88.9 | 0.063 | 3.66 |
| Li-Co-MBGNs | -112.5 | 23.3 | -103.8 | 76.7 |  |  | 3.23 |

*Dc*, degree of condensation.

**Table S6 Nanoparticle textural properties**

| BGNs | Surface Area (m²/g) | Pore Volume (cm³/g) | Pore Size (Å) |
| --- | --- | --- | --- |
| uMBGNs | 656 | 0.49 | 42 |
| Li-MBGNs | 650 | 0.47 | 37 |
| Co-MBGNs | 605 | 0.43 | 39 |
| Li-Co-MBGNs | 602 | 0.43 | 38 |

**References:**

X. Ding, J. Zheng, F. Ju, L. Wang, J. Kong, J. Feng and T. Liu: Facile fabrication of hollow mesoporous bioactive glass spheres: From structural behaviour to in vitro biology evaluation. *Ceram. Int.*, 47(24), 34836-34844 (2021) doi:10.1016/j.ceramint.2021.09.024

W. Song, Z. Jin, X. Huang, Z. Xi, X. Luo and L. Cen: Microfluidic-preparation of PLGA microcarriers with collagen patches for MSCs expansion and osteogenic differentiation. *Eur. Polym. J.*, 170, 111177 (2022) doi:https://doi.org/10.1016/j.eurpolymj.2022.111177

D. Zhu, B. Lu, Q. Yang, H. Yu, P. Liu, J. Yin, Y. Chen, Y. Huang, Q. Ke, C. Zhang, Y. Guo and Y. Gao: Lanthanum-doped mesoporous bioglasses/chitosan composite scaffolds enhance synchronous osteogenesis and angiogenesis for augmented osseous regeneration. *Chem. Eng. J.*, 405, 127077 (2021) doi:https://doi.org/10.1016/j.cej.2020.127077

Y. Wu, Y. Wang and X. Nabi: Protective effect of Ziziphora clinopodioides flavonoids against H2O2-induced oxidative stress in HUVEC cells. *Biomed. Pharmacother.*, 117, 109156 (2019) doi:https://doi.org/10.1016/j.biopha.2019.109156

P. Han, C. Wu, J. Chang and Y. Xiao: The cementogenic differentiation of periodontal ligament cells via the activation of Wnt/β-catenin signalling pathway by Li+ ions released from bioactive scaffolds. *Biomaterials*, 33(27), 6370-6379 (2012) doi:10.1016/j.biomaterials.2012.05.061
